# Effects of Living Conditions, Subjective Integration, and Social Networks on Health-Related Quality of Life among the Migrant Elderly Following Children in Jinan, China

**DOI:** 10.3390/healthcare9040414

**Published:** 2021-04-02

**Authors:** Tingting Tian, Fanlei Kong, Shixue Li

**Affiliations:** 1 Centre for Health Management and Policy Research, School of Public Health, Cheeloo College of Medicine, Shandong University, Jinan 250012, China; tianfamily123@163.com; 2 NHC Key Lab of Health Economics and Policy Research, Shandong University, Jinan 250012, China

**Keywords:** migrant elderly following children, health-related quality of life, living conditions, subjective integration, social networks

## Abstract

With accelerated urbanization in China, an increasing number of the migrant elderly following children (MEFC) have appeared. This study aims to explore the effects of living conditions, subjective integration, and social networks on the health-related quality of life (HRQOL) of MEFC in Jinan, China. HRQOL was assessed by the 12-item Short-Form Health Survey, which included the mental component summary (MCS) and the physical component summary (PCS). Univariate analyses and binary logistic regression were used to investigate the association between the above indicators and HRQOL. A total of 656 MEFC were selected by multi-stage cluster random sampling, 25.2% and 25.0% of whom were defined as poor MCS and poor PCS, respectively. Those who understood the local dialect, could trust others, and connected with friends were more likely to have good MCS; those with a nanny, faulty elevator, and no support from their spouse were the reverse. MEFC who were trans-city, had no elevator or a faulty elevator, and went to the hospital alone were more likely to have poor PCS; those who approved of living conditions in their hometowns were the reverse. Results indicated that better living conditions, stronger subjective integration, and wider social networks led to higher HRQOL of MEFC. Implications of the government, communities, and families of MEFC were given to improve their HRQOL.

## 1. Introduction

Over the past decades, with the rapid development of China’s economy and the general improvement in medical technology, the process of aging and urbanization has gradually intensified, and the scale of population mobility has been constantly expanding [1]. From 1990 to 2015, the number of internal migrant populations increased from 21.35 million to 247 million [2]. Influenced by Confucian culture, the elderly in China traditionally migrated to a new place to take care of their grandchildren and reunite with their family [3]; they have come to be referred to as the migrant elderly following children (MEFC). As a result, the number of the migrant elderly reached 13.04 million in 2015, accounting for 5.3% of the total migrant population [2]. This redistribution of the elderly population brought some public health challenges, such as poor health outcomes [4], transmission of infectious diseases [5,6], inequalities in using essential public health services [7], and excessive growth of urban medical expenses [8]. Consequently, the health status of MEFC is a key issue worthy of attention.

Health-related quality of life (HRQOL) is defined as an individual’s satisfaction or happiness with the quality of their physical and mental health and is deemed a comprehensive assessment tool of a population’s health status [9]. It was found that elderly Iranian migrants in Sweden had a worse HRQOL compared to Swedish natives [10]. A lower level of HRQOL was also observed in Turkish, Moroccan, and Moluccan elderly in the Netherlands [11]. However, most of the research on migrants’ HRQOL in China have focused on workers. Rural-to-urban migrant workers with diabetes reported significantly worse HRQOL [12]. Whereas perceived work-related stress, feelings of depression, and poor sleep had a negative impact on HRQOL of white-collar migrant workers [13].

Previous research clarified that living conditions were determinants of health [14]. A population-based study in Chile reported that migrants living with poor household materials would have poorer health status [15]. Another study on recently arrived migrants in Sweden demonstrated that living in crowded houses increased the risk of suffering from mental illness [16]. Organista et al. [17] found that difficult living conditions were related to psychological distress in Latino migrant day laborers, but better social networks with family could moderate this relationship. In China, a study by Ding et al. [18] showed that migrant pregnant women living in rented houses were more likely to experience childbirth in their hometowns. In addition, Dou et al. [19] explained that housing conditions were a reason for migration among elderly adults, but they did not explore housing conditions’ relationship with elderly adults’ HRQOL.

Social integration has been proven to be one of the most important issues affecting the physical and mental health of the migrant elderly [20]. It is a multidimensional concept with no unified definition at present. For migrants, many scholars have adopted different measurement systems that could be divided into two main dimensions: objective integration, including economic integration and behavioral adaptation, and subjective integration, including acculturation and self-identity [21]. Several studies have indicated that subjective integration has an impact on the health status of migrants [22]. Adedeji et al. [23] found that subjective integration was positively associated with the physical and psychological health of sub-Saharan African migrants in Germany. Victor’s [24] results showed that place identity as an aspect of self-identity was positively related to HRQOL of African migrants in Australia. A study among migrants in Shanghai illustrated that subjectivity was an important factor in reducing migrants’ psychological pressure [25]. However, to date, there have been few studies on MEFC as the research object.

Social networks could be described as social units [26] or the structure of social ties and the web of social relationships that surround a particular individual or group [27]. People who have closer and more diverse social networks tend to be healthier and more positive [28]. Social networks from family and friendship relations are main sources for the elderly in their later life [29]. Studies showed that poor social support from family and intergenerational relationships were associated with a worse mental state among the elderly [30]. For the migrant elderly, formal and informal communication networks influenced their use of modern check-up equipment [31] and their probability of utilizing health care [32]. Additionally, frequent contact and living with a partner or children were able to reduce the loneliness of the migrant elderly and protect them from mental illness [33]. Another study found that elderly Asian migrants in New Zealand living alone or only with their spouses were more likely to feel isolated [34]. However, social networks of the migrant elderly were generally unable to meet their needs because of the busy lives of social network members [35]. To date, limited studies have explored the role of social networks in HRQOL of MEFC in China.

Given the above background, there is no study that simultaneously explores the effects of living conditions, subjective integration, and social networks on HRQOL, let alone takes MEFC as the research object. Thus, this study aims to explore the effects of living conditions, subjective integration, and social networks on HRQOL of MEFC in Jinan, China.

## 2. Materials and Methods

### 2.1. Data and Sample

The data were collected in Jinan City, Shandong Province, China, in August 2020. Shandong Province lies in the east of China, and Jinan City is the capital city of Shandong Province. Its Gross Domestic Product (GDP) in 2020 was 1.01 trillion Chinese Yuan (≈157,285.51 million USD) [36]. As of 1 July 2020, Jinan had 10 districts and 2 counties (132 sub-districts and 29 towns) under its jurisdiction [37]. By the end of 2019, the local resident population was 8.91 million, while the registered population was 7.98 million [38]. There were 2.9 million migrants in Jinan in 2019 [39]; those who were older than 60 years and followed their children to Jinan were the subjects of this study. Multi-stage cluster random sampling was used to select the participants.

In the first stage of the data collection, three districts were chosen from the 10 districts as the primary sampling units (PSUs), considering their economic development and geographic location. In the second stage, a total of three sub-districts were selected from each of the PSUs as the secondary sampling units (SSUs). In the third stage, three communities were selected from each of the SSUs. All the migrants who were older than 60 years and followed their children to Jinan in these three communities constituted the total sample of this study.

In total, 32 university students became investigators for this study after receiving training regarding the background information of the study, contents of the questionnaire, and techniques of social survey. The investigators conducted approximately twenty-minute face-to-face interviews with the subjects to collect the data. A total of 670 MEFC were initially selected and interviewed. However, 14 of them were excluded due to obvious logical errors in their responses or uncompleted questionnaires. A total of 656 participants were ultimately included in the study.

### 2.2. Measurements

#### 2.2.1. Health-Related Quality of Life (HRQOL)

The Short-Form Health Survey (SF-12), consisting of 12 items drawn from the eight subscales of the SF-36 [40], is widely used to measure HRQOL [41] and has been tested as a scale with good reliability and validity [42]. The Chinese-translated version of the SF-12 questionnaire has been reported to be suitable for the elderly in China [43,44]. In this study, HRQOL of MEFC was assessed by SF-12 scores that were divided into the mental component summary (MCS) and the physical component summary (PCS) scores for calculation [45]. Each participant’s MCS and PCS were dichotomized by the cut-off point of the first quartile of MCS and PCS scores, and we defined poor HRQOL as scores lower than that of the first quartile [46,47].

#### 2.2.2. Social Demographic Characteristics

Social demographic characteristics, which were collected as a basis for comparison, included age, gender, marital status, education, duration in inflow area, hiring a nanny, and migration space range.

#### 2.2.3. Living Conditions

Living conditions were captured by three indicators, including two objective indicators of current residence, “type of housing” and “elevator faults”, and one subjective evaluation of “living conditions in hometown.”

#### 2.2.4. Subjective Integration

Subjective integration was mainly measured through the dimensions of acculturation and self-identity. Factors including “local dialect” and “trust others around you” were used to measure the dimension of acculturation. Self-identity was measured by the question “Do you feel like a local?”

#### 2.2.5. Social Networks

Social networks in this study were measured through two dimensions: family relationships and social relationships. Participants’ family relationships were measured by their responses to four questions: “Do you have different consumption values from your children?” “Do your children work overtime at home because of their busy schedule?” “Have you gone to the hospital without children’s company for fear of troubling them?” “Do you have support from your spouse?” Social relationships were measured by two indicators: “number of local friends” and “relationships with friends and colleagues.”

### 2.3. Statistical Analysis

All statistical analyses were performed using IBM SPSS 24.0, and *p*-values less than 0.05 were regarded as statistically significant. Chi-square test was used to calculate the differences in MCS and PCS among the subgroups of each categorical variable. After univariate analyses, statistically significant variables were included in the logistic regression analyses. Three binary logistic regression models with an enter method were adopted to explore the associations between independent variables and HRQOL. Meanwhile, crude odds ratios (OR) and 95% confidence intervals (95% CI) were calculated. First, social demographic characteristics and living conditions entered Model 1, then the indicators of subjective integration were brought into Model 2, and finally the variables of social networks were added to Model 3.

## 3. Results

### 3.1. Participant Characteristics

The basic information of social demographic characteristics, living conditions, subjective integration, and social networks of 656 participants is provided in Table 1. It was shown that 57.9% of the sample was 63 to 68 years old. Most respondents were female (63.7%), married (88.9%), had an education level of junior school and below (81.1%), did not hire a nanny (99.1%), stayed in the inflow area for less than six years (66.0%), and were trans-city migrants (67.2%). As for living conditions, 83.5% of the participants lived in high-rise residential buildings, 28.8% had no elevator in their houses, and more than half of them (50.5%) provided a good evaluation of living conditions in their hometowns. With regard to subjective integration, nearly 50% of MEFC said that there were different degrees of dialect barriers, 39.2% did not feel like a local, and less than 10% rarely or never trusted others around them. In terms of social networks, most participants had local friends (86.1%) and support from their spouse (82.8%), frequently contacted friends and colleagues (45.3%), had no different consumption values from their children (58.1%), and had never gone to the hospital without children’s company (58.4%). Additionally, 58.1% of the children of MEFC never worked overtime at home.

### 3.2. HRQOL in the Mental and Physical Dimensions

As shown in Table 1, factors that were significantly different in MCS included hiring a nanny (*p* < 0.05), type of housing (*p* < 0.05), elevator faults (*p* < 0.001), local dialect (*p* < 0.01), trusting others (*p* < 0.05), support from their spouse (*p* < 0.05), and relationships with friends and colleagues (*p* < 0.05). As for PCS, there were statistically significant differences in age (*p* < 0.05), migration space range (*p* < 0.01), type of housing (*p* < 0.001), elevator faults (*p* < 0.001), living conditions in their hometowns (*p* < 0.05), local dialect (*p* < 0.05), feeling like a local (*p* < 0.05), different consumption values from children (*p* < 0.05), children working overtime at home (*p* < 0.01), going to the hospital alone (*p* < 0.01), support from their spouse (*p* < 0.05), number of local friends (*p* < 0.05), and relationships with friends and colleagues (*p* < 0.05).

### 3.3. Relationship between Independent Variables and HRQOL in the Mental Domains

Table 2 and Table 3 display the *p*-values, OR, and 95% CI for the association of the significant variables after univariate analyses with MCS and PCS, respectively. The collinearity diagnostics’ results revealed that the tolerances of all independent variables were much greater than 0.1, and the variance inflation factors were far less than 10, suggesting that there was no multicollinearity between independent variables in three logistic regression models.

In Table 2, the results of Model 1 indicate that hiring a nanny and elevator faults were significant factors of MCS. When variables of subjective integration entered Model 2, these two variables were still statistically significant. Local dialect and rarely trusting others emerged as statistically significant. In Model 3, the Hosmer and Lemeshow test showed the *p*-value (*p* = 0.449) was greater than 0.05 and the percentage accuracy in classification was 75.8%, indicating a high prediction effect. Model 3 presented that living conditions, subjective integration, and social networks were statistically associated with MCS. Specifically, MEFC who often had elevator faults (*p* = 0.008, OR = 0.135), were able to understand or speak local dialect (*p* < 0.01, OR < 1), could trust others (*p* = 0.023, OR = 0.087), and kept frequent contact with friends and colleagues (*p* = 0.035, OR = 0.492) were more likely to have good MCS, but for those who hired a nanny (*p* = 0.035, OR = 6.476), sometimes had elevator faults (*p* = 0.042, OR = 1.657), and had no support from their spouse (*p* = 0.039, OR = 1.826), the scores were reversed.

### 3.4. Relationship between Independent Variables and HRQOL in the Physical Domains

As shown in Table 3, migration space range and elevator faults were significant predictors of PCS in Model 1. However, as subjective integration was added to Model 2, the significance of migration space range disappeared. With a *p*-value greater than 0.05 in the Hosmer and Lemeshow test and 81.7% accuracy in classification, the results of Model 3 indicated that migration space range, living conditions, and social networks were significantly associated with PCS. Specifically, the elderly who were trans-city migrants (*p* = 0.040, OR = 1.860), had no elevator in their houses (*p* < 0.001, OR = 5.922) or sometimes had elevator faults (*p* = 0.029, OR = 1.988), and had sometimes (*p* = 0.049, OR = 1.832) or often (*p* = 0.031, OR = 2.265) gone to the hospital alone were more likely to suffer from poor PCS, and those who gave a good (*p* = 0.010, OR = 0.316) or fair (*p* = 0.025, OR = 0.341) evaluation of living conditions in their hometowns were more likely to have good PCS.

## 4. Discussion

### 4.1. HRQOL Profile of MEFC in Jinan, China

In this study, the mean scores of MCS and PCS were 55.44 ± 7.10 and 48.51 ± 9.59, respectively. Compared with community-dwelling elderly in Zhejiang, China [48], the MCS scores of MEFC were higher and PCS scores were similar. This may be because family reunions are beneficial to the mental health of the migrant elderly [49].

### 4.2. Effects of Independent Variables on HRQOL of MEFC

#### 4.2.1. Association between Living Conditions and HRQOL

The present study demonstrated that living conditions were associated with HRQOL of MEFC. Some studies have suggested that recreational amenities [50], home amenities, and community safety [51] are influencing factors in migrants’ HRQOL. The absence of an elevator or an occasional faulty elevator in the residence would not only cause adverse effects on the physical health of MEFC [52], but also increase anxiety for their own safety. However, the results showed that frequent elevator faults were associated with better MCS. A possible reason could be that it typically took a long time for the rural elderly to use the infrastructure and electronic devices in the city, which might be embarrassing for them [53]. The elevator faults gave them an excuse to attribute their slowness to faulty equipment, which would make them feel relaxed. Another reason might be that the elderly would not want to use elevators with very frequent faults, in order to avoid being frightened or injured. Furthermore, relatively good evaluations of living conditions in their hometowns were proven to be a protective factor of PCS. Somewhat dissimilar from our findings, a previous study reported that the stronger the hometown identity, the lower the life satisfaction of Chinese rural migrants [54]. Nevertheless, when we considered the traditional concept of the missing hometown of most Chinese elderly people [55], our results were understandable. For the elderly who migrated to a new city following their children, the house in their hometown served not only as an accommodation when they visited their relatives during the traditional festivals, but also as a kind of emotional sustenance. Hence, better living conditions in migrants’ hometowns may contribute to a better health status for MEFC.

#### 4.2.2. Association between Subjective Integration and HRQOL

This study demonstrated that the impact of subjective integration on HRQOL of MEFC was greater on the mental aspect (MCS) through the dimension of acculturation, compared with the physical aspect (PCS). Language barriers have always been one of the most difficult issues affecting the normal work and life of migrants in the inflow area [56,57,58]. Moreover, China has a variety of traditional cultures and dialects. Although Mandarin is a common language in China, there are still obstacles for the majority of MEFC to communicate smoothly with the local people. Similarly, those who could not understand or speak the local dialect reported the poorest MCS in our study because they were forced to stay at home and unable to connect with their peers around them. Additionally, we found that trusting others had a significant positive influence on MCS. Numerous studies illustrated this point: less trust in others was directly associated with poorer self-rated health status of Mexican-Americans in Texas [59]; distrust reduced the quality of life of rural-to-urban migrants [60]; and compared with the local urban elderly, the migrant elderly reported lower trust and a higher risk of mental illness [61]. Thus, helping MEFC establish a sense of trust with others is one of the keys to improving their HRQOL.

#### 4.2.3. Association between Social Networks and HRQOL

Social networks have been found to be associated with HRQOL. Having support from spouses and close relationships with friends and colleagues demonstrated significant differences in MCS. According to other studies, spouses may be able to provide stability and emotional comfort for the migrant elderly [62] and may make a vital contribution to developing healthy behavioral habits [63]. High levels of social activity with friends decreased negative effects on the well-being of older adults [64], and both the quality and frequency of contact with friends were strongly related [65]. Therefore, as new members of a community, harmonious relations with their spouses and local friends could help MEFC become a part of the inflow area, which could not be replaced by a good relationship with their children. Moreover, we took MEFC’s experience of going to the hospital without their children’s company for fear of troubling them as one of the indicators to assess their family relationships in this study. As indicated above, this kind of experience had a negative impact on their PCS, because the original health status of the elderly with medical needs might be relatively low. In addition, falls have been one of the main factors threatening old people’s health, and often occur in public places [66]. It is generally known that the company of children could reduce the possibility of elderly falls.

#### 4.2.4. Association between Social Demographic Characteristics and HRQOL

In terms of demographic characteristics, the significant predictors of HRQOL were hiring a nanny at home and migration space range. Hiring a nanny was a risk factor for MCS of MEFC, probably because the elderly and nannies had different views on taking care of their grandchildren. Nannies usually receive professional training and adopt more scientific methods, while the elderly tend to follow their traditional experiences [67]. Regarding migration space range, the elderly with trans-city migration had a lower PCS than those who migrated across the county, which was consistent with previous research results. Long-distance migration would bring greater risks and more losses to the elderly; the migrant elderly had to leave their familiar physical environment, cultural atmosphere, and interpersonal relationships [68,69]. The wholeness of their daily lives changed dramatically and was more challenging, which was harmful to their HRQOL.

### 4.3. Implications

To improve the HRQOL of MEFC, the government may take the following measures. First, on the basis of protecting the diversity of traditional culture, continue to promote the popularization of Mandarin to alleviate the obstacles of dialect among the elderly, vigorously promote the construction of an elderly-friendly city, and reinforce the safety of public facilities so that the elderly can live in a safe and convenient social environment. Second, as the central living space of MEFC, the community plays an important role in improving their HRQOL and is supposed to conduct more recreational activities, so as to increase opportunities for MEFC to build close friendships and trust with the local residents. Meanwhile, it is an essential responsibility of the community to maintain the security of its infrastructure. Last but not least, the children of MEFC should accompany and be filial toward their parents in order to create a happy family atmosphere.

### 4.4. Limitations

There are several limitations to this study. First, we used data from a cross-sectional study, so the causal relationship could not be predicted. Second, due to the lack of a systematic scale on living conditions, subjective integration, and social networks in the questionnaire, we only selected some relevant indicators to conduct our evaluation, which was expected to be remedied in the follow-up study. Finally, affected by COVID-19, our study failed to conduct the survey in Shanghai as planned and only selected MEFC in Jinan as the research object, which might not represent HRQOL of MEFC in China.

## 5. Conclusions

In summary, to the best of our knowledge, this was the first study to explore the determinants of HRQOL among Chinese MEFC from the perspective of living conditions, subjective integration, and social networks. The results indicated that the better the living conditions, the stronger the subjective integration, and the wider the social networks, the higher the HRQOL of MEFC. Understanding the local dialect, trusting others, and maintaining contact with friends positively impacted MCS, but hiring a nanny, sometimes having elevator faults, and having no support from their spouse impacted MCS negatively. Trans-city migration, elevator faults, and going to the hospital alone had negative effects on PCS, and better evaluations of living conditions in their hometowns reversed this effect.

## Figures and Tables

**Table 1:** Univariate analyses for HRQOL of MEFC in the mental and physical dimensions.

Variables	Total	Good MCS	Poor MCS	*p*	Good PCS	Poor PCS	*p*
	*n* (%)	*n* (%)	*n* (%)		*n* (%)	*n* (%)	
**Gender**				0.831			0.639
Male	238 (36.3)	177 (74.4)	61 (25.6)		176 (73.9)	62 (26.1)	
Female	418 (63.7)	314 (75.1)	104 (24.9)		316 (75.6)	102 (24.4)	
**Age (years)**				0.782			0.015
60–62	126 (19.2)	91 (72.2)	35 (27.8)		88 (69.8)	38 (30.2)	
63–65	197 (30.0)	144 (73.1)	53 (26.9)		149 (75.6)	48 (24.4)	
66–68	183 (27.9)	139 (76.0)	44 (24.0)		137 (74.9)	46 (25.1)	
69–71	87 (13.3)	70 (80.5)	17 (19.5)		69 (79.3)	18 (20.7)	
72–79	49 (7.5)	36 (73.5)	13 (26.5)		43 (87.8)	6 (12.2)	
80–	14 (2.1)	11 (78.6)	3 (21.4)		6 (42.9)	8 (57.1)	
**Marital status**				0.855			0.830
Married	583 (88.9)	437 (75.0)	146 (25.0)		438 (75.1)	145 (24.9)	
Divorced/Widowed	73 (11.1)	54 (74.0)	19 (26.0)		54 (74.0)	19 (26.0)	
**Education**				0.739			0.324
Unschooled	196 (29.9)	143 (73.0)	53 (27.0)		147 (75.0)	49 (25.0)	
Primary school	144 (22.0)	109 (75.7)	35 (24.3)		110 (76.4)	34 (23.6)	
Junior school	192 (29.3)	142 (74.0)	50 (26.0)		136 (70.8)	56 (29.2)	
Senior school or above	124 (18.9)	97 (78.2)	27 (21.8)		99 (79.8)	25 (20.2)	
**Hire a nanny**				0.038			0.636
No	650 (99.1)	489 (75.2)	161 (24.8)		487 (74.9)	163 (25.1)	
Yes	6 (0.9)	2 (33.3)	4 (66.7)		5 (83.3)	1 (16.7)	
**Duration in inflow area**				0.117			0.623
≤3 years	223 (34.0)	175 (78.5)	48 (21.5)		167 (74.9)	56 (25.1)	
3–6 years	210 (32.0)	147 (70.0)	63 (30.0)		162 (77.1)	48 (22.9)	
>6 years	223 (34.0)	169 (75.8)	54 (24.2)		163 (73.1)	60 (26.9)	
**Migration space range**				0.146			0.006
Trans-county	146 (22.3)	119 (81.5)	27 (18.5)		124 (84.9)	22 (15.1)	
Trans-city	441 (67.2)	321 (72.8)	120 (27.2)		315 (71.4)	126 (28.6)	
Trans-provincial	66 (10.1)	49 (74.2)	17 (25.8)		50 (75.8)	16 (24.2)	
Transnational	3 (0.5)	2 (66.7)	1 (33.3)		3 (100.0)	0 (0.0)	
**Type of housing**				0.032			<0.001
Low-rise residence	108 (16.5)	72 (66.7)	36 (33.3)		61 (56.5)	47 (43.5)	
High-rise residence	548 (83.5)	419 (76.5)	129 (23.5)		431 (78.6)	117 (21.4)	
**Elevator faults**				<0.001			<0.001
Never	171 (26.1)	136 (79.5)	35 (20.5)		152 (88.9)	19 (11.1)	
Sometimes	235 (35.8)	164 (69.8)	71 (30.2)		188 (80.0)	47 (20.0)	
Often	61 (9.3)	59 (96.7)	2 (3.3)		52 (85.2)	9 (14.8)	
No elevator	189 (28.8)	132 (69.8)	57 (30.2)		100 (52.9)	89 (47.1)	
**Living conditions in hometown**				0.489			0.015
Very poor	34 (5.2)	28 (82.4)	6 (17.6)		20 (58.8)	14 (41.2)	
Poor	19 (2.9)	13 (68.4)	6 (31.6)		14 (73.7)	5 (26.3)	
Fair	115 (17.5)	87 (75.7)	28 (24.3)		88 (76.5)	27 (23.5)	
Good	331 (50.5)	240 (72.5)	91 (27.5)		263 (79.5)	68 (20.5)	
Very good	157 (23.9)	123 (78.3)	34 (21.7)		107 (68.2)	50 (31.8)	
**Local dialect**				0.006			0.011
Cannot understand or speak	30 (4.6)	15 (50.0)	15 (50.0)		21 (70.0)	9 (30.0)	
Understand but cannot speak	293 (44.7)	218 (74.4)	75 (25.6)		208 (71.0)	85 (29.0)	
Basic mastery	170 (25.9)	127 (74.7)	43 (25.3)		125 (73.5)	45 (26.5)	
Mastery	163 (24.8)	131 (80.4)	32 (19.6)		138 (84.7)	25 (15.3)	
**Feel like a local**				0.216			0.033
Totally disagree	64 (9.8)	50 (78.1)	14 (21.9)		48 (75.0)	16 (25.0)	
Somewhat disagree	193 (29.4)	135 (69.9)	58 (30.1)		133 (68.9)	60 (31.1)	
Somewhat agree	262 (39.9)	197 (75.2)	65 (24.8)		197 (75.2)	65 (24.8)	
Totally agree	137 (20.9)	109 (79.6)	28 (20.4)		114 (83.2)	23 (16.8)	
**Trust others around you**				0.024			0.156
Never	6 (0.9)	3 (50.0)	3 (50.0)		2 (33.3)	4 (66.7)	
Rarely	44 (6.7)	40 (90.9)	4 (9.1)		33 (75.0)	11 (25.0)	
Sometimes	207 (31.6)	152 (73.4)	55 (26.6)		158 (76.3)	49 (23.7)	
Often	399 (60.8)	296 (74.2)	103 (25.8)		299 (74.9)	100 (25.1)	
**Different consumption values from children**				0.484			0.010
No	475 (72.4)	359 (75.6)	116 (24.4)		369 (77.7)	106 (22.3)	
Yes	181 (27.6)	132 (72.9)	49 (27.1)		123 (68.0)	58 (32.0)	
**Children work overtime at home**				0.447			0.002
Never	381 (58.1)	279 (73.2)	102 (26.8)		297 (78.0)	84 (22.0)	
Rarely	109 (16.6)	88 (80.7)	21 (19.3)		66 (60.6)	43 (39.4)	
Sometimes	67 (10.2)	51 (76.1)	16 (23.9)		53 (79.1)	14 (20.9)	
Often	99 (15.1)	73 (73.7)	26 (26.3)		76 (76.8)	23 (23.2)	
**Go to the hospital alone**				0.308			0.003
Never	383 (58.4)	291 (76.0)	92 (24.0)		306 (79.9)	77 (20.1)	
Rarely	139 (21.2)	108 (77.7)	31 (22.3)		100 (71.9)	39 (28.1)	
Sometimes	82 (12.5)	56 (68.3)	26 (31.7)		54 (65.9)	28 (34.1)	
Often	52 (7.9)	36 (69.2)	16 (30.8)		32 (61.5)	20 (38.5)	
**Support from spouse**				0.013			0.049
Yes	543 (82.8)	396 (72.9)	147 (27.1)		399 (73.5)	144 (26.5)	
No	113 (17.2)	95 (84.1)	18 (15.9)		93 (82.3)	20 (17.7)	
**Number of local friends**				0.728			0.010
0	91 (13.9)	67 (73.6)	24 (26.4)		68 (74.7)	23 (25.3)	
1–2	195 (29.7)	145 (74.4)	50 (25.6)		130 (66.7)	65 (33.3)	
3–5	177 (27.0)	129 (72.9)	48 (27.1)		139 (78.5)	38 (21.5)	
6–	193 (29.4)	150 (77.7)	43 (22.3)		155 (80.3)	38 (19.7)	
**Relationships with friends and colleagues**				0.010			0.015
Never contact	67 (10.2)	44 (65.7)	23 (34.3)		45 (67.2)	22 (32.8)	
Little contact	123 (18.8)	89 (72.4)	34 (27.6)		85 (69.1)	38 (30.9)	
Occasional contact	169 (25.8)	118 (69.8)	51 (30.2)		122 (72.2)	47 (27.8)	
Frequent contact	297 (45.3)	240 (80.8)	57 (19.2)		240 (80.8)	57 (19.2)	
**Total**	656 (100.0)	491 (74.8)	165 (25.2)		492 (75.0)	164 (25.0)	

Notes: HRQOL = health-related quality of life, MEFC = migrant elderly following children, MCS = mental component summary, PCS = physical component summary.

**Table 2:** Binary logistic regression for relationships between related variables and MCS of MEFC.

Variables	Model 1			Model 2			Model 3		
	Demography + Living Conditions			Model 1 + Subjective Integration			Model 2 + Social Networks		
	*p*	OR	95% CI	*p*	OR	95% CI	*p*	OR	95% CI
**Hire a nanny**									
No		1.0			1.0			1.0	
Yes	0.049	5.612	1.008–31.260	0.042	6.074	1.071–34.435	0.035	6.476	1.143–36.699
**Type of housing**									
Low-rise residence		1.0			1.0			1.0	
High-rise residence	0.106	0.653	0.389–1.096	0.062	0.605	0.357–1.026	0.103	0.640	0.373–1.095
**Elevator faults**									
Never		1.0			1.0			1.0	
Sometimes	0.029	1.679	1.053–2.678	0.022	1.755	1.086–2.834	0.042	1.657	1.019–2.694
Often	0.007	0.133	0.031–0.572	0.005	0.123	0.028–0.538	0.008	0.135	0.031–0.592
No elevator	0.197	1.416	0.835–2.402	0.220	1.405	0.816–2.419	0.260	1.370	0.792–2.371
**Local dialect**									
Cannot understand or speak					1.0			1.0	
Understand but cannot speak				0.003	0.295	0.132–0.662	0.005	0.300	0.131–0.691
Basic mastery				0.003	0.280	0.120–0.650	0.006	0.294	0.123–0.701
Mastery				<0.001	0.215	0.091–0.509	0.001	0.236	0.097–0.573
**Trust others around you**									
Never					1.0			1.0	
Rarely				0.021	0.090	0.012–0.699	0.023	0.087	0.011–0.717
Sometimes				0.248	0.347	0.058–2.087	0.254	0.337	0.052–2.187
Often				0.262	0.361	0.061–2.140	0.331	0.399	0.062–2.546
**Support from spouse**									
Yes								1.0	
No							0.039	1.826	1.030–3.237
**Relationships with friends and colleagues**									
Never contact								1.0	
Little contact							0.239	0.660	0.330–1.317
Occasional contact							0.734	0.892	0.460–1.728
Frequent contact							0.035	0.492	0.255–0.951

Notes: MCS = mental component summary, MEFC = migrant elderly following children, OR = crude odds ratios, 95% CI = 95% confidence intervals.

**Table 3:** Binary logistic regression for relationships between related variables and PCS of MEFC.

Variables	Model 1			Model 2			Model 3		
	Demography + Living Conditions			Model 1 + Subjective Integration			Model 2 + Social Networks		
	*p*	OR	95% CI	*p*	OR	95% CI	*p*	OR	95% CI
**Age(years)**									
60–62		1.0			1.0			1.0	
63–65	0.223	0.709	0.408–1.233	0.198	0.688	0.389–1.216	0.253	0.707	0.390–1.281
66–68	0.303	0.748	0.431–1.299	0.309	0.747	0.426–1.311	0.350	0.758	0.423–1.356
69–71	0.285	0.685	0.342–1.370	0.251	0.663	0.328–1.338	0.311	0.683	0.327–1.428
72–79	0.051	0.374	0.139–1.005	0.080	0.408	0.149–1.115	0.184	0.492	0.173–1.399
80–	0.154	2.459	0.715–8.464	0.140	2.656	0.725–9.721	0.077	3.331	0.879–12.621
**Migration space range**									
Trans-county		1.0			1.0			1.0	
Trans-city	0.007	2.103	1.231–3.595	0.055	1.733	0.988–3.040	0.040	1.860	1.029–3.361
Trans-provincial	0.076	2.029	0.928–4.436	0.258	1.598	0.709–3.603	0.206	1.727	0.740–4.032
Transnational	0.999	0.000	0.000–	0.999	0.000	0.000–	0.999	0.000	0.000–
**Type of housing**									
Low-rise residence		1.0			1.0			1.0	
High-rise residence	0.335	0.773	0.458–1.305	0.345	0.773	0.453–1.319	0.461	0.808	0.459–1.423
**Elevator faults**									
Never		1.0			1.0			1.0	
Sometimes	0.009	2.213	1.221–4.010	0.013	2.144	1.177–3.907	0.029	1.988	1.073–3.683
Often	0.730	1.168	0.483–2.825	0.570	1.296	0.529–3.177	0.362	1.538	0.609–3.884
No elevator	<0.001	6.370	3.456–11.740	<0.001	6.461	3.484–11.981	<0.001	5.922	3.073–11.411
**Living conditions in hometown**									
Very poor		1.0			1.0			1.0	
Poor	0.337	0.523	0.139–1.963	0.319	0.508	0.135–1.921	0.073	0.284	0.072–1.127
Fair	0.195	0.553	0.226–1.355	0.109	0.477	0.193–1.179	0.025	0.341	0.133–0.876
Good	0.101	0.503	0.222–1.143	0.059	0.451	0.197–1.029	0.010	0.316	0.132–0.757
Very good	0.788	1.124	0.479–2.636	0.986	0.993	0.421–2.342	0.511	0.741	0.303–1.811
**Local dialect**									
Cannot understand or speak					1.0			1.0	
Understand but cannot speak				0.781	0.870	0.326–2.322	0.783	0.867	0.313–2.397
Basic mastery				0.947	0.966	0.345–2.706	0.936	0.957	0.328–2.790
Mastery				0.226	0.518	0.179–1.504	0.267	0.533	0.176–1.618
**Feel like a local**									
Totally disagree					1.0			1.0	
Somewhat disagree				0.625	1.199	0.578–2.486	0.897	0.949	0.434–2.078
Somewhat agree				0.601	0.824	0.398–1.704	0.468	0.748	0.341–1.638
Totally agree				0.300	0.642	0.277–1.485	0.469	0.717	0.291–1.766
**Different consumption values from children**									
No								1.0	
Yes							0.459	1.208	0.733–1.991
**Children work overtime at home**									
Never								1.0	
Rarely							0.069	1.667	0.961–2.891
Sometimes							0.471	0.764	0.367–1.590
Often							0.762	0.910	0.495–1.674
**Go to the hospital alone**									
Never								1.0	
Rarely							0.865	1.054	0.574–1.938
Sometimes							0.049	1.832	1.003–3.349
Often							0.031	2.265	1.079–4.754
**Support from spouse**									
Yes								1.0	
No							0.064	1.858	0.964–3.584
**Number of local friends**									
0								1.0	
1–2							0.812	0.919	0.456–1.850
3–5							0.276	0.663	0.316–1.390
6–							0.257	0.654	0.314–1.364
**Relationships with friends and colleagues**									
Never contact								1.0	
Little contact							0.660	1.190	0.549–2.578
Occasional contact							0.256	1.545	0.729–3.272
Frequent contact							0.929	0.967	0.458–2.043

Notes: PCS = physical component summary, MEFC = migrant elderly following children, OR = crude odds ratios, 95% CI = 95% confidence intervals.

## Data Availability

The data are available on request from the corresponding author in this study. The data are not publicly available due to privacy restrictions.
